
JOURNAL INFORMATION
==============================
NLM Title Abbreviation: Med Chir Rev
NLM Journal ID: 0034617

The Medico-Chirurgical Review

Publisher: Biomedical Journal Digitization Project

ARTICLE INFORMATION
==============================
PMCID: PMC4966262
PMID: 29917632
Article version: 1
Subjects: Reviews, Diseases Produced by Malaria

Surgical Observations on the Treatment of Chronic Inflammation in Various Structures, &c.

Print publication date: 1828 Oct 1
Volume: 9
Issue: 18
First page: 307
Last page: 310
1828 Jul
License: This work is free of known copyright restrictions. For more information, please see PMC Back Issue Digitization.
License URL: https://creativecommons.org/publicdomain/mark/1.0/

==============================

author will give us credit for. Nothing but a strong conviction that the work before us contains a multitude of valuable gems, which readers in general will not take the trouble to pick out, could have induced us to bestow so much labour on a review, at this season of the year, (June 1828,) when the town is so full of men and malaria, that our literary labours only *commence* when "church-yards yawn," and all our other brethren (accoucheurs excepted) are fast asleep! In rendering Dr. Macculloch's work more accessible to the profession, by diffusing it in a portable and perusable shape, we are conscious that we are doing the state some service—and the author no injury. We have several other articles from the same work in reserve.

---

II.

*Surgical Observations on the Treatment of Chronic Inflammation in various Structures; particularly as exemplified in the Diseases of the Joints.* By JOHN SCOTT, Surgeon to the London Ophthalmic Infirmary; and Assistant Surgeon to the London Hospital. Octavo, pp. 291. 1828.

IT was our intention to have noticed this work at the time of its first appearance, and with that view we then perused and examined it with some attention. By some accident, however, the copy prepared for review was mislaid; and the subject in consequence was overlooked and forgotten, until chance again, within these few days, presented the stray volume to our hands. But it is now, we fear, too late to pursue our original design; for the work no longer possesses any interest on the score of novelty, nor are its intrinsic merits such as to entitle it at this late period to an extended examination here. Under these circumstances, we shall, on the present occasion, confine ourselves within very narrow limits; our object being now, not to analyse the work itself, or expatiate on the subjects of which it treats, but simply to discharge a duty we have long imposed upon ourselves, that namely, of placing on record in these pages every cotemporary statement or suggestion, which seems calculated to be of any practical utility in the art of medicine.

1. *On the Treatment of Ulcers on the Legs.*

It is well known to most of our readers that the mode of treatment introduced some years ago by Mr. Baynton, of Bristol, for the cure of ulcers on the legs, viz. by *adhesive straps*, has never been found so successful in the hands of others, as it was stated to have been originally in his. Occurrences of this kind, however, are too common in the practice of physic to excite much surprise, and too easily accounted for on general principles to demand much investigation. Mr. Baynton's mode of treatment, therefore, has gradually been suffered to fall almost into disuse; and practitioners in the present day are generally inclined to believe that its merits were much overstated by him, or that there was "an art and mystery" in its original application, which he did not, and perhaps could not, by mere description, communicate to others. Mr. Scott, however, a respectable practitioner at Bromley in Kent,* seems to have thought rather differently on this subject, and appears, in consequence, to have applied himself to modify and improve upon the plan of Mr. Baynton, instead of rejecting or neglecting it altogether, as others had done. The result of this has been, the adoption by this gentleman of a mode of treatment in chronic ulcers of the legs, which will be found, we are strongly inclined to believe, on many occasions, superior to that of Mr. Baynton—but on this point the reader may determine for himself, after perusing the following statement.

When an ulcerated leg is treated according to Mr. Baynton's method, it is surrounded, as the reader is aware, by straps of adhesive plaster, applied in a circular manner over a space extending from about one inch below, to about two inches above the ulcerated part. The whole limb, then, from the toe to the knee, is swathed in a spiral roller; and as the object is to *compress* as well as *support* the parts, both this and the adhesive straps are drawn around the limb with as much force as the patient can conveniently bear.

By this mode of proceeding, however, an equal and uniform degree of support and compression never can be applied to the whole limb—for the part wrapped in *adhesive plaster* must always be subjected to a greater degree of pressure, and that more constantly applied than the parts which are surrounded by the roller alone. Hence pain and swelling and inflammation often arise in a limb so treated, which compel us for a time to suspend our proceedings, or perhaps force us to abandon them altogether.

To obviate these inconveniences Mr. Scott proposes, 1st, that the whole limb should be equally and uniformly covered with adhesive plaster, as well as with the roller—and 2ndly, that both the straps and the roller should be applied with such a degree of force, only, as may be sufficient to support the parts steadily, without compressing them into a smaller compass, as recommended by Mr. Baynton. "With regard to the method of fulfilling these indications" the following directions are given by our author.

"The Emplastrum Plumbi, spread on calico, is the best application, as it does not irritate the skin.† It is most conveniently made use of when cut into slips of fifteen inches in length by two in breadth.

"The foot being placed at a right angle to the leg, one of the slips should be ap-

* We say Mr. Scott of Bromley, for it is with him we believe, and not with the author of the work before us, (who is his son,) that the plan we are about to notice originated.—*Rev.*

† An effect, we may observe, which the plaster made use or recommended by Mr. Baynton often did produce, in consequence of the quantity of *resin* it contained. —*Rev.*

plied from the first bone of the great toe, along the inner edge of the foot, around the posterior part of the os calcis to the first bone of the little toe; the middle of another slip should then be placed under the bottom of the os calcis, and its ends extended perpendicularly up on each side of the leg; the third is to be applied along the foot, parallel to the first, and overlapping the half of it; the fourth should be placed parallel to the second, overlapping the half of it, and extending perpendicularly up the sides of the leg. In this manner they (the slips) should be applied alternately along the foot, and up the leg, the one holding and as it were antagonizing the other in the motions of the foot until the whole limb is covered from the toes to the knee. Subsequently to this, a calico bandage is applied in the usual manner, first alternately around the foot and ankle, and then up the leg as high as the knee.

"It is necessary to be particularly careful that the plasters and bandage be applied in such a manner that their superior and inferior edges are accurately placed in apposition to the skin, otherwise they will exert an unequal pressure, which is highly injurious. The whole should be applied with only that degree of tightness which is perfectly agreeable to the feelings of the patient, and not with a view of compressing the parts into a smaller space. In this manner every vessel in the limb will be uniformly and effectually supported.

"In respect to the time at which it will be necessary to renew the applications, that must be regulated by the quantity of the discharge, for when applied in the manner that has been described, they will remain for weeks, or even for months, without altering their position in the least." 15.

### 2. On the Treatment of Chronic Enlargements of the Joints.

It sometimes happens that certain joints in the body are attacked with pain and stiffness, and swelling, of a character so indolent, as scarcely to excite any constitutional disturbance, yet, at the same time, so obstinate as to resist all our ordinary modes of treatment. Under these circumstances the disease, of which these symptoms form a part, continues in general its progress onwards, slowly indeed but steadily, until all the parts and tissues of which the affected joint consists, are involved in one destructive process, and a state of general irritation is produced which threatens the patient's life.*

To avert this danger the affected limb must often about this time be removed altogether—or such other measures perhaps be adopted as will lead, if successful, to the permanent extinction of all motion in the joint.

Death, then, or mutilation, or a stiffened joint are the only terminations to which, generally speaking, we look forward in the present day in cases of disease such as that now under consideration. Mr. Scott, however, assures us that his father, the gentleman already alluded to in this article, has long been in the habit of treating such cases with a very different result; and professes, in the work before us, to make known a plan of treatment, for such affections,

* The knee, ankle, elbow, and wrist, are the joints most usually attacked by this disease; which, from the appearance of the part affected is vulgarly denominated *white swelling*, and by the learned, from one of its usual effects or symptoms, *arthropyosis*, that is to say, *a joint* with *pus* in it—αρθρον—πυον.—*Rev.*

"the efficacy of which," he says, "I have seen verified in numerous cases, first, under the care of my father, and since under my own."

This plan consists in the simultaneous and steady application to the part affected, of moderate pressure, and a camphorated mercurial ointment—under the combined influence of which, as we are assured, the pain and stiffness, and swelling, will in general gradually disappear, and all the parts return to a healthy state. The details relative to this plan we now proceed to give in our author's own words, premising merely that the application of leeches will sometimes be necessary as a preliminary measure in cases where symptoms of active inflammation exist.

"In the first place, the surface of the joint, suppose the knee, is to be carefully cleansed by a sponge, soft brown soap and warm water, and then thoroughly dried; next, this surface is to be rubbed by a sponge soaked in camphorated spirit of wine, and this is continued a minute or two, until it begins to feel warm, smarts somewhat, and looks red. It is now covered with a soft cerate made with equal parts of the Ceratum Saponis and Unguentum Hydrargyri fortius cum Camphorâ.* This is thickly spread on large square pieces of lint, and applied entirely around the joint, extending for at least six inches, above and below the point at which the condyles of the femur are opposed to the head of the tibia; over this, to the same extent, the limb is to be uniformly supported by strips of calico, spread with the emplastrum plumbi of the London Pharmacopœia. These strips are about one inch and a half broad, and vary in length; some are fifteen inches, others a foot, others half these two lengths, and the shorter or longer are selected according to the size of the part round which they are to be applied."

"Over this adhesive bandage, thus applied, comes an additional covering of emplastrum saponis, spread on thick leather, and cut into four broad pieces, one for the front, the other for the back, the two others for the sides of the joint. Lastly, the whole is secured by means of a calico bandage, which is put on very gently, and rather for the purpose of securing the plaster, and giving greater thickness and security to the whole, than for the purpose of compressing the joint. This is an important point, as otherwise an application which almost invariably affords security and ease, may occasion pain, with all its attendant mischief.

"In some cases, in which the skin is thick and indolent, sufficient irritation will scarcely be excited by the above applications, and this may be promoted by rubbing on a small quantity of tartar emetic ointment previously to the application of the cerate. This, however, is rarely necessary.

"In some cases, also, it is desirable more effectually to prevent the motion of the limb, particularly in children. This may be done, by applying on each side of the joint, externally to the plasters, a piece of pasteboard, softened by soaking in water, and cut into the length, breadth, and form of splints. These being soft, will accommodate themselves to the figure of the joint, and, when dry, effectually preclude all motion."

"The remedies thus applied will not require very frequent removal. The time during which they may be left undisturbed, will depend chiefly on the necessity for a repetition of the bleeding, in which we must be guided by the degree of pain, or when there are open abscesses, by the quantity of the discharge. Should neither of these influence the question, the only necessity for removing the dressings will arise from their having ceased to keep up any irritation in the skin. In some cases it will be necessary to re-apply them every week; in the generality of instances they may be allowed to remain a fortnight, and in others for a longer time. Even where there are open wounds, I allow them to remain several days, or a week, being firmly convinced by experience that the presence of the matter does less harm than the frequent disturbance of the part. A strumous ulcer can scarcely be disturbed too seldom; nothing does so much harm as officious dressing and probing." 139.

* The author has not told us any where the relative quantity of camphor employed by him or his father in this composition, which is not officinal.—*Rev.*
